# Mobile Phone App for Self-Monitoring of Eating Rhythm: Field Experiment

**DOI:** 10.2196/11490

**Published:** 2019-03-27

**Authors:** Saara Pentikäinen, Hannu Tanner, Leila Karhunen, Marjukka Kolehmainen, Kaisa Poutanen, Kyösti Pennanen

**Affiliations:** 1 VTT Technical Research Centre of Finland Ltd Espoo Finland; 2 VTT Technical Research Centre of Finland Ltd Oulu Finland; 3 Institute of Public Health and Clinical Nutrition Department of Clinical Nutrition University of Eastern Finland Kuopio Finland

**Keywords:** mHealth, behavior observation, self-regulation, eating, ecological momentary assessment

## Abstract

**Background:**

Temporal aspects of eating are an integral part of healthy eating, and regular eating has been associated with good diet quality and more successful weight control. Unfortunately, irregular eating is becoming more common. Self-monitoring of behavior has been found to be an efficient behavioral change technique, but the solution should be simple enough to ensure long-lasting adherence.

**Objective:**

This study aimed to explore the influence of self-monitoring of daily eating pattern with mobile phone app on eating rhythm, eating behavior tendencies, and the underlying motives and attitudes related to eating.

**Methods:**

A mobile phone app, *Button,* was developed for effortless self-monitoring of eating rhythm. The feasibility of the app was tested in a 30-day intervention. The participants (N=74) recorded their eating occasions during the intervention by pressing a button in the app widget.

**Results:**

The average interval between meals increased (96 [SD 24] min during the first 10 days vs 109.1[SD 36.4] during the last 10 days) and the number of daily eating occasions decreased (4.9 [SD 0.9] during the first 10 days vs 4.4 [SD 0.9] during the last 10 days). The tendencies for cognitive restraint, emotional eating, and uncontrolled eating increased. Eating-related attitudes and motives remained largely unchanged.

**Conclusions:**

These results indicate that a simple self-monitoring tool is able to draw a user’s attention to eating and is a potential tool to aid people to change their eating rhythm.

## Introduction

Eating rhythm is an integral part of healthy eating. A regular eating pattern including breakfast, lunch, dinner, and 1 to 2 snacks has been found to be associated with good diet quality [1], whereas skipping breakfast has been consistently found to associate with poor overall diet quality and exposure to weight gain [2,3]. In addition, eating less than 3 times a day negatively influences appetite control, and unplanned snacking and consumption of the major part of the energy at the end of the day seem unfavorable for weight balance [4,5]. On the other hand, a recent review was not able to confirm associations between eating frequency and body weight [6]. Nevertheless, irregular eating has been associated with various adverse health effects [7] as it may complicate weight regulation via hindered circadian system [8]. A recent review also found irregular eating habits to be associated with increased risk of metabolic syndrome and cardiometabolic risk factors [9].

Reports from different parts of the world suggest that irregular eating is becoming more common. Irregular eating patterns that manifest as a tendency to skip conventional meals (*unsynchronized eating patterns*) have increased in Nordic countries; approximately one-fifth of Danish, Finnish, Norwegian, and Swedish people have been found to possess unsynchronized eating patterns during weekdays and about one-third during weekends [10]. The prevalence is specifically high among young and singles. Similarly, a meal pattern with obscured meal times was found to be more common in young German adults than in older adults [11]. Irregular eating patterns and vast differences between weekdays and weekends have also been observed in US adults; breakfast-lunch-dinner pattern has been found to be largely absent and the fasting period (night fast) has been found to be relatively short [12]. In addition, snacking has increased [13]. Moreover, approximately one-fourth of Australian adults have been found to follow a *grazing* pattern in which there are no clear meal times but frequent peaks of eating occasions during the day [14]. Therefore, actions are needed to change the course toward a more regular, health-supporting eating rhythm.

Changing habits requires well-developed self-regulation, which in turn is enabled by self-monitoring and self-evaluation of progress [15]. Self-monitoring, which assists individuals to become aware of their current behavior, has been successfully applied in weight-loss interventions using both traditional methods as well as self-monitoring with mobile apps [16]. Moreover, adherence to weight management intervention has been found to be better with mobile phone-based intervention compared with website or paper food diary based–interventions [17]. An array of mobile apps related to nutrition are launched yearly and installed by millions of people [18]. A majority of the apps are food diaries that provide detailed dietary information when they are consistently filled in. However, food diaries are laborious for the user and they might suffer from problems with memory and interpretation of the data. Therefore, an ecological momentary assessment (EMA) where events are recorded in real time in the natural environment has also been suggested as a tool for nutrition research to collect more accurate information about dietary behavior and underlying reasons for the behaviors [19]. EMA builds a picture of an individual’s habits by recording multiple days. For example, EMA has been recently used to evaluate how fasting influences disordered eating behaviors [20] and if the meal and snack-time eating disorder cognitions predict eating disorder behavior [21]. The previous apps utilized EMA as a methodological tool to collect data on a moment of the behavior or occurrence, which was the focus of a study. EMA approaches could also be applied in the context of behavior change, as is the case of this study. Self-monitoring of eating rhythm offers a possibility to direct users’ attention to their own eating patterns and push them toward positive behavioral changes with low burden on the user.

The EMA tool (mobile app *Button*) was developed for the self-monitoring of eating rhythm in real time. At present, there are apps available (eg, in Google Play) reminding about eating times (eg, Meal Reminder) or coaching fasting (eg, BodyFast Intermittent Fasting) for certain time periods. Unlike the current apps, the *Button* app does not remind or coach the user to eat but makes eating rhythm visible and thus grants the ownership of eating rhythm to the user. The idea of the app is to offer the user a simple tool to become aware of his or her temporal eating pattern, which might act as a stimulus to regularize eating.

The aim of the study was to explore the influence of self-monitoring of daily eating pattern with an EMA mobile app on eating rhythm, eating behavior tendencies, and underlying motives and attitudes related to eating. A further aim was to study whether 1 of the 2 app versions (healthy-unhealthy dichotomy or content-discontent dichotomy) is more influential.

## Methods

### Ecological Momentary Assessment Mobile App Development

**Figure 1:**
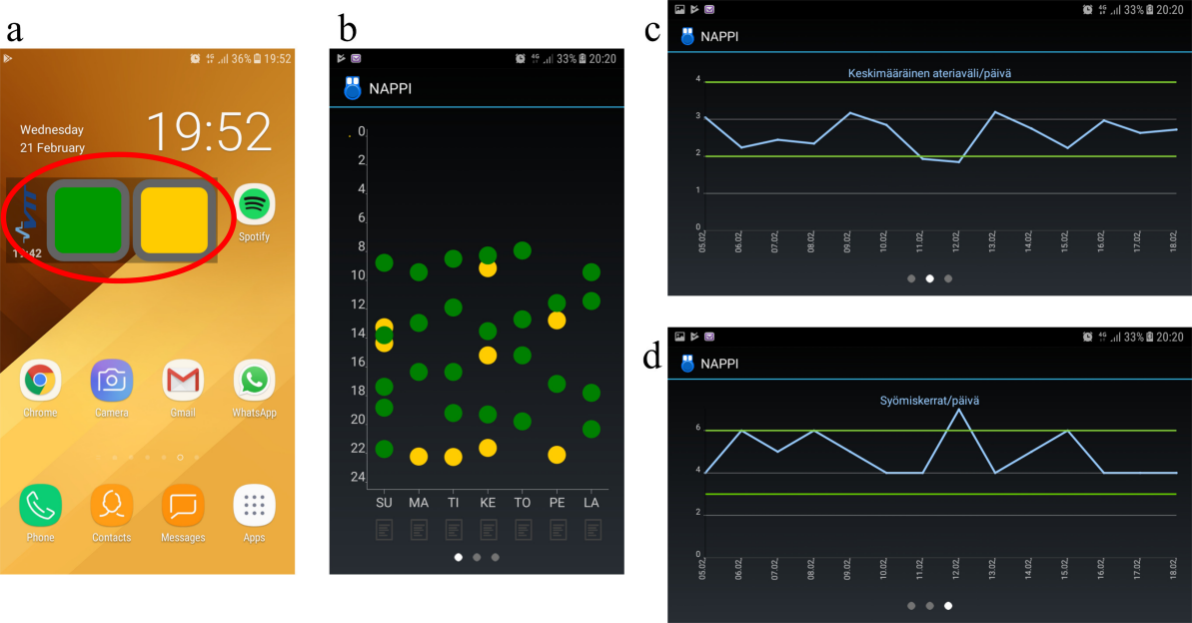
The Button widget with green and yellow buttons (circled) on mobile phone desktop (a) and visual summaries shown in the Button application (b-d). The user presses either green (content with eating occasion) or yellow (discontent with eating occasion) button of the widget after every eating occasion. The first visualization (b) in the application shows the eating occasions during the past seven days (weekdays on x-axis, time on y-axis), the second screen (c) shows the average interval between eating occasions per day during the past 14 days and, the third screen (d) shows the average number of eating occasions per day during the past 14 days. Green horizontal lines in c and d indicate the shortest (2 h) and longest (4 h) recommended interval between eating occasions and the smallest (3) and the highest (6) recommended number of eating occasions per day.

The Button comprises 2 components: the desktop widget and the actual app. The user presses 1 of the 2 buttons reflecting different types of eating occasions (healthy or unhealthy or content or discontent) in the Button widget after each eating occasion to record the time stamp and type of the eating occasion. The Button app visualizes the user’s eating pattern with 3 summary screens (Figure 1). The data on the user’s eating occasions are automatically transferred to a research database, where user data are protected using identification codes and encryption. The app frontend was implemented for Android mobile devices using Java, whereas the server backend utilizes Spring framework.

The app development was an iterative process including 2 real-life user trials in January 2017 and April 2017. The aim of the user trials was to find a feasible way to record the eating occasions to guarantee the technical functionality of the Button app and easiness to use. Volunteers (trial 1:9 and trial 2:8) used the app for a 2-week period, which was followed by a focus group discussion about the usability aspects and recommendations for further development. The participants of the first trial found it nebulous to interpret the graphs related to the optimal interval between meals and number of eating occasions per day. Therefore, green horizontal lines depicting the recommendations (2- to 4-hour intervals among meals, 3-6 meals per day) were included in the app after the first trial. This change increased the easiness of the data interpretation in the second trial.

### Two App Versions

The user trial participants found color coding in the Button widget as the most feasible option to differentiate eating occasions. They also preferred the healthy (green)-unhealthy (red) dichotomy for the buttons. Considering that healthiness is not the only viewpoint when evaluating eating and food choices, a version with content (green)-discontent (yellow) dichotomy was also developed. It was considered that the possibility to make a subjective judgment about whether the eating episode is subjectively good or bad versus normative healthy-unhealthy logic might provide the user with a stronger feeling of autonomy and commitment and therefore motivate the user to change eating habits [22-25]. Thus, 2 app versions (*Healthiness* version and *Contentment* version) were developed. The *Healthiness* version had green and red buttons, the green button meaning an eating occasion that the user perceived as healthy and the red button meaning an occasion that was perceived as unhealthy. The *Contentment* version had green and yellow buttons. The green button reflected an eating occasion that the user was content with and the yellow button reflected an eating occasion the user was not fully content with. Only the meanings for the 2 buttons of the widget varied, but the functionalities of the 2 app versions were the same.

### Self-Monitoring Study

Participants (n=74) were recruited through public advertisements, email advertisements, and institutions’ intranets in 2 university campus areas in Finland. The participants had to be over 18 years of age and interested in well-being. In addition, an updated Android phone (Version 4.3 or newer) was a prerequisite for the attendance. The volunteers with red-green color blindness (self-reported) were excluded from the study.

The eligible volunteers were invited to the study location where the details of the study were explained, and they had a chance to ask questions. After receiving both written and verbal information about the study (voluntariness, purpose, content, and confidentiality), the volunteers signed an informed consent form. The Button app was installed in the participants’ personal mobile phones, and they were instructed on how to use the app. The participants were given 4 movie tickets worth 52 euros to compensate their time and effort. Data collection was conducted in May and June 2017. The study protocol was approved by the Coordinating Research Ethics Committee of the Helsinki and Uusimaa Hospital District. The study was conducted according to the ethical principles of good research and clinical practice described in the Declaration of Helsinki.

The participants were randomly distributed to *Healthiness* group or *Contentment* group. Background information about the participants is given in Table 1. The members of the Healthiness and Contentment groups were alike regarding gender distribution, age, body mass index (BMI), and perceived importance of new technologies. The share of participants currently working was higher in the Healthiness group than in the Contentment group. There were 20 normal weight, 13 overweight, and 4 obese in the Healthiness group and 14 normal weight, 15 overweight, and 8 obese in the Contentment group.

**Table 1:** Background information about the participants.

Variables	All participants (N=74)	Healthiness group (n=37)	Contentment group (n=37)	t test (df)	Chi-square test (df)	P value
Gender, number of females, n (%)	45 (61)	24 (65)	21 (57)	—^a^	0.5 (1)	.48
Age (years), mean (SD)	36.2 (12.5)	35.4 (11.2)	36.9 (13.8)	0.518 (72)	—	.14
Body mass index (kg/m^2^), mean (SD)	26.1 (4.9)	25.5 (4.9)	26.7 (4.8)	1.026 (72)	—	.99
Work situation, number of participants working currently, n (%)	67 (91)	36 (97)	31 (84)	—	3.9 (1)	.05
Personal importance of new technologies^b^, mean (SD)	5.2 (1.4)	5.1 (1.4)	5.2 (1.3)	0.294 (72)	—	.70

^a^Not applicable.

^b^measured on a 7-point scale in which 1=not important at all and 7=extremely important.

**Figure 2:**
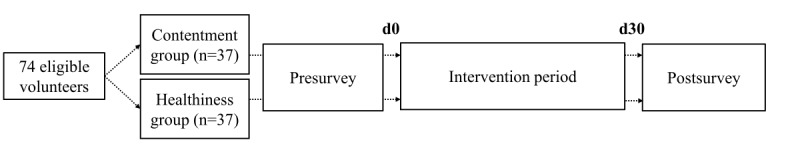
Study design.

### Study Design and Experimental Procedure

The study adopted within- and between-subject design with 2 independent study groups using 1 of the 2 app versions (*Healthiness* group and *Contentment* group, Figure 2).

The participants were instructed to record the eating occasions (excluding eating occasions with calorie-free drinks only) by pressing either green or red (*Healthiness* group) or green or yellow (*Contentment* group) buttons after eating according to their own evaluation about the eating occasion. No instructions were given about eating rhythm or dietary choices. The participants were also instructed that they could freely open the Button app and observe the visual summaries of their eating rhythm and add comments. The intervention lasted for 30 days. Surveys measuring participants’ self-reported daily meals, attitudinal constructs, food choice motives, and eating behavior tendencies were administered before and after the intervention period to detect potential changes caused by the Button usage.

### Independent Variables

Table 2 presents the independent variables used in this study, their reliability, and sources variables (Cronbach alpha values higher than .70 are considered sufficient [26]). The main interest was in the changes in the eating rhythm during the intervention period. Therefore, the main independent variables were derived from the Button press data. The data were sent automatically from the Button app in the participants’ mobile phone to the research database. The surveys before (presurvey) and after the intervention (postsurvey) comprised variables related to the eating rhythm (daily consumed meals), of eating behavior tendencies—the three-factor eating questionnaire (TFEQ)—, discontent with eating, attitudes toward health (general health interest), and relevant food choice-related motives (health, mood, and weight control). Daily consumed meals were measured by asking the respondent to mark down the meals that he or she consumes daily. The meal options included breakfast, lunch, afternoon snack, dinner or such, evening snack, and 5 unspecified options. The number of daily consumed meals was calculated as a sum of the marked meals. The TFEQ was administered to assess the potential effects of self-monitoring of eating pattern on the eating behavior tendencies [27]. A recently modified version (TFEQ-R15) of the questionnaire was used [28]. All items were measured with 4-point scales. The raw scale scores were transformed to a 0 to 100 scale ([raw score–lowest possible raw score]/highest possible raw score×100) [29]. The higher raw scale scores mean greater tendency toward the measured subscale. Discontent with eating at different meal times was measured by asking the respondents to rate the frequency of discontent at different meal times (breakfast, lunch time, afternoon, dinner time, late evening or in the night, and some other time) on a scale of 1=never to 5=very often. The mean value for discontent in each meal time was calculated.

**Table 2:** Reliability and sources of independent variables (Cronbach alpha values higher than .70 are considered sufficient).

Category		Content	Cronbach alpha presurvey	Cronbach alpha postsurvey	Source
**Button data**					
	Eating rhythm	Interval between eating occasions per day	—^a^	—	Button app
		Eating occasions per day	—	—	Button app
	Adherence to use the app	App openings per day to observe visual summaries about eating rhythm	—	—	Button app
**Survey data**					
	Eating rhythm	Daily consumed meals	—	—	List of meals types
	Eating behavior tendencies	Three-factor eating questionnaire (R-15) (scale 0-100)	uncontrolled eating: .77; cognitive restraint: .76; emotional eating: .77	uncontrolled eating: .81; cognitive restraint: .71; emotional eating: .89	[27,28]
	Discontent with eating	Discontent with eating in different meal times (scale 1=never, 5=very often)	—	—	modified from [28]
	Attitudes	General health interest (scale: 1=completely disagree, 7=completely agree)	.81	.80	[30]
	Food-related motives	Food choice motives regarding health, mood, weight control (subscales of food choice questionnaire (scale: 1=not important at all, 4=very important)	health: .75; mood: .80; weight control: .76	health: .74; mood: .88; weight control: .79	[31]

^a^Not applicable.

Postsurvey also included an open-ended question about gained insights related to one’s personal eating rhythm, eating behavior, and factors affecting these. First, participants were asked if they gained insights about their eating habits during the intervention period. In a positive case, the participant was asked to describe those insights in writing.

### Data Analysis

Complete case analysis was conducted. The analysis included those participants (n=59) who completed the 30-day intervention successfully. Those participants (n=15) who had more than 4 days per 1 of the 3 10-day periods without any stamps because of technical difficulties or incompliance were removed from the Button data. The analytical approach was chosen as the Button app is intended to be used voluntarily and thus those participants who continued to use the app during the entire study were deemed to represent the real-life user. Pre and postsurvey data were analyzed on an intention-to-treat basis including all the 74 participants in the analyses. A different analytical approach was applied as all users had been exposed to Button usage at least to some extent during the entire 30-day period. Therefore, the survey data from the participants with and without valid Button data were deemed comparable.

The collected Button data were preprocessed to screen out faulty data and to prepare them for the actual analysis. Records deemed as duplicate values (more than 1 timestamp within 5 min) were removed, and timestamp values were converted from server time to actual local time. To determine the actual number of eating occasions per day, it was decided that the day begins and ends at 4 am instead of midnight, and the data were handled accordingly. Before the analysis, data were treated in the following manner: (1) for the evaluation of the changes in eating rhythm, the 30-day intervention period was divided into 3 10-day periods, (2) all days with less than an average of 1,000 seconds (<17 min) interval between the meals were removed (197/1770 days, 11%) as this was considered as an indication of multiple miss-presses or a technical flaw; moreover, evident outlier days with only 1 or 2 stamps per day were removed, (3) after suspicious data were removed, an average was calculated for each participant for each 10-day period, and the average was applied for the days with missing data.

SPSS Statistics (Version 24, IBM Corp, Chicago, IL, USA) was used for the statistical analysis. Repeated measures analysis of variance (ANOVA) with Bonferroni adjustment for multiple comparisons was used to analyze the within-group changes in eating rhythm (interval between meals, number of eating occasions per day) and adherence (number of app openings per day; n=59). A 1-way ANOVA was carried out to study between-group differences in data derived from the Button app (interval between eating occasions, number of eating occasions per day, and adherence to use the app). False discovery rate (FDR) was controlled by using the Benjamini-Hochberg method. The survey data analyses were conducted for all the participants (N=74) and separately for the subgroups. Repeated-measure ANOVA with Bonferroni adjustment for multiple comparisons were conducted to analyze within-group changes between presurvey and postsurvey. A 1-way ANOVA was used to analyze between-group differences in the self-reported number of daily meals, eating behavior tendencies (TFEQ-R15), discontent with eating habits, general health interest, and food-related motives. Benjamini-Hochberg method was used to control the FDR.

Responses to the open-ended question about insights gained during the intervention period were analyzed following the standard content analysis procedures. Reported individual insights were categorized on the basis of their content in appropriate higher-order subcategories and finally in main categories.

## Results

### Eating Rhythm

#### Interval Between Eating Occasions

**Figure 3:**
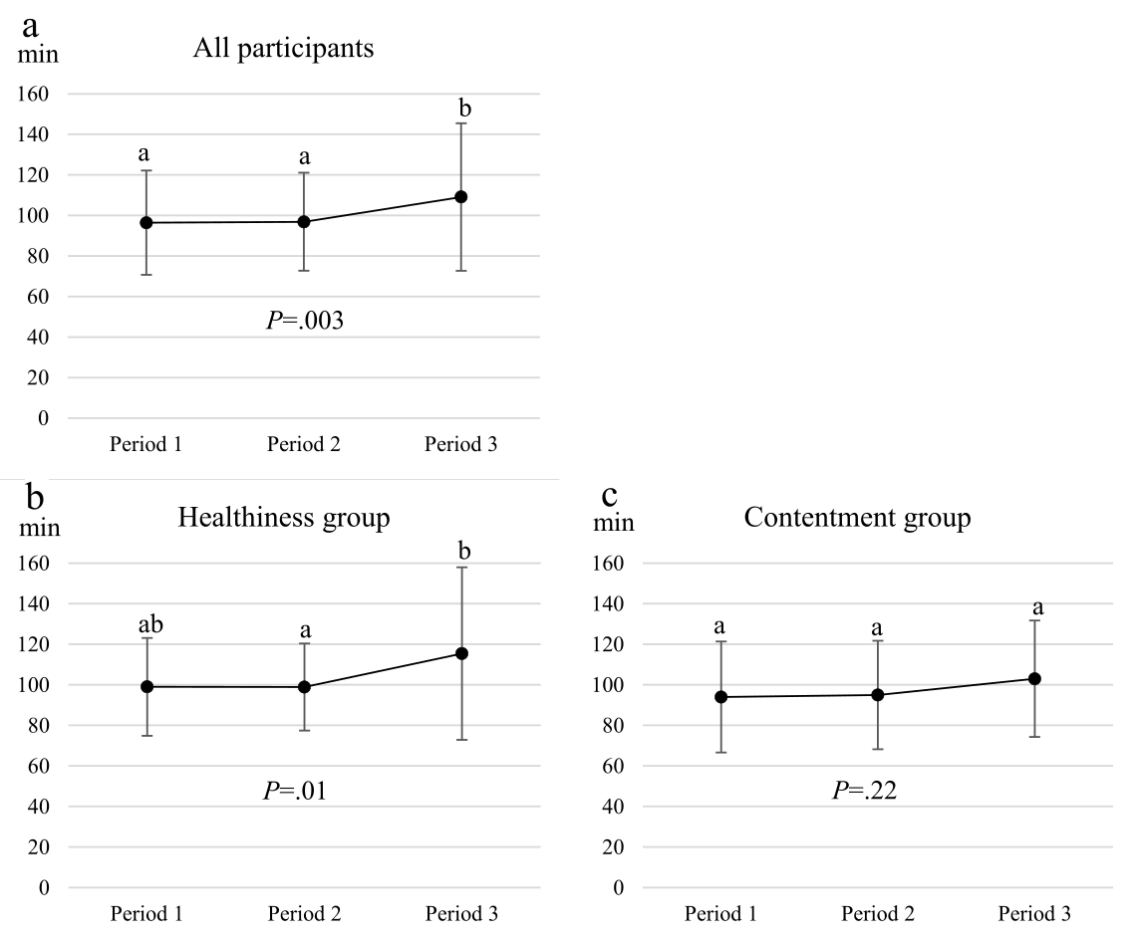
Average intervals between eating occasions (Button presses) (mean ± SD) in the three periods (Period 1 = days 1-10, Period 2 = days 11-20, Period 3 = days 21-30) in a) all participants (n=59) and members of b) Healthiness group (n=29) and c) Contentment group (n=30). Different superscript letters indicate a statistically significant difference (P≤.05) between study periods.

The average interval between eating occasions (on the basis of button presses) was 96±24 min in all participants during the first 10 days of intervention, and it increased to 109.1±36.4 min (*F*_*1,694*_=6.241, *P*=.003) during the last 10 days of the intervention (Figure 3). This was mainly because of the increased interval between eating occasions in the Healthiness group between the last 2 study periods (*P*=.03). The average interval between eating occasions did not vary in the Contentment group. Between-group analyses revealed no statistically significant differences in any of the periods (Period 1: *F*_*1*_=.549, *P*=.46; Period 2: *F*_*1*_=.389, *P*=.54; Period 3: *F*_*1*_=1.734, *P*=.19).

#### Number of Meals Per Day

In the presurvey, participants reported to consume approximately 4.5±0.9 meals per day (Table 3). There were no differences between the groups (*F*_*1*_=.825, *P*=.37). The number of the reported daily meals was significantly lower after the intervention in all participants (4.2[SD 1.0] meals; *t*_*73*_=2.591, *P*=.01) and again mainly caused by the change in Healthiness group. There were no statistically significant differences in the number of daily meals between the groups after the intervention (*F*_*1*_=.015, *P*=.90).

**Figure 4:**
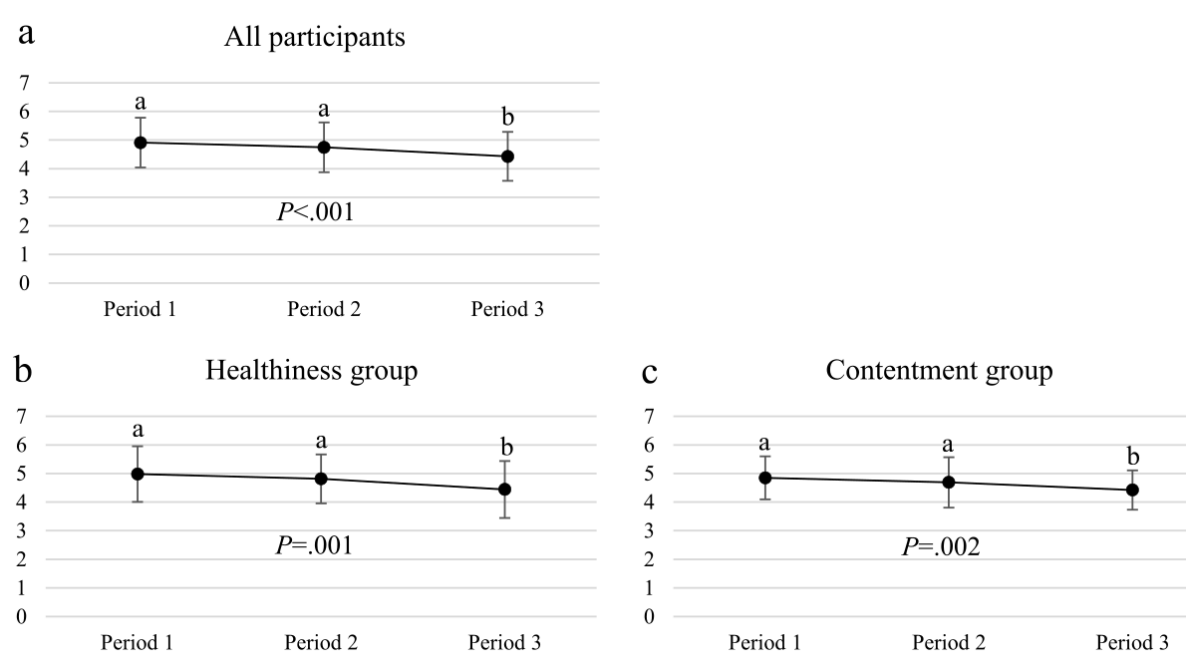
Average number of reported eating occasions per day (mean ± SD) in the three periods (Period 1 = days 1-10, Period 2 = days 11-20, Period 3 = days 21-30) in a) all participants (n=59) and members of b) Healthiness group (n=29) and c) Contentment group (n=30). Different superscript letters indicate a statistically significant difference between study periods.

The number of reported eating occasions (button presses) reduced during the 4-week intervention among all participants (Figure 4). The trend was similar in both the Healthiness group and Contentment group, and the groups did not differ from each other (Period 1: *F*_*1*_=.333, *P=*.57; Period 2: *F*_*1*_=.289, *P=*.59; and Period 3: *F*_*1*_=.007, *P=*.93). On average, 77% of the eating occasions of the Healthiness group were classified as *healthy* and 86% of the eating occasions of the Contentment group were classified as *content*.

**Table 3:** The number of reported meals (breakfast, lunch, afternoon snack, dinner, evening snack, and other snacks) per day (mean [SD]) before and after the intervention in (1) all participants (N=74) and members of (2) Healthiness group (n=37), and (3) Contentment group (n=37).

Meals per day	Mean (SD)		*F* test (*df*)	*P* value
	Before	After		
All participants (N=74)	4.5 (0.9)	4.2 (1.0)	6.712 (1)	.01
Healthiness group (n=37)	4.6 (0.8)	4.2 (1.1)	4.776 (1)	.04
Contentment group (n=37)	4.4 (1.0)	4.2 (0.9)	1.974 (1)	.17

### Adherence to Use the App

**Figure 5:**
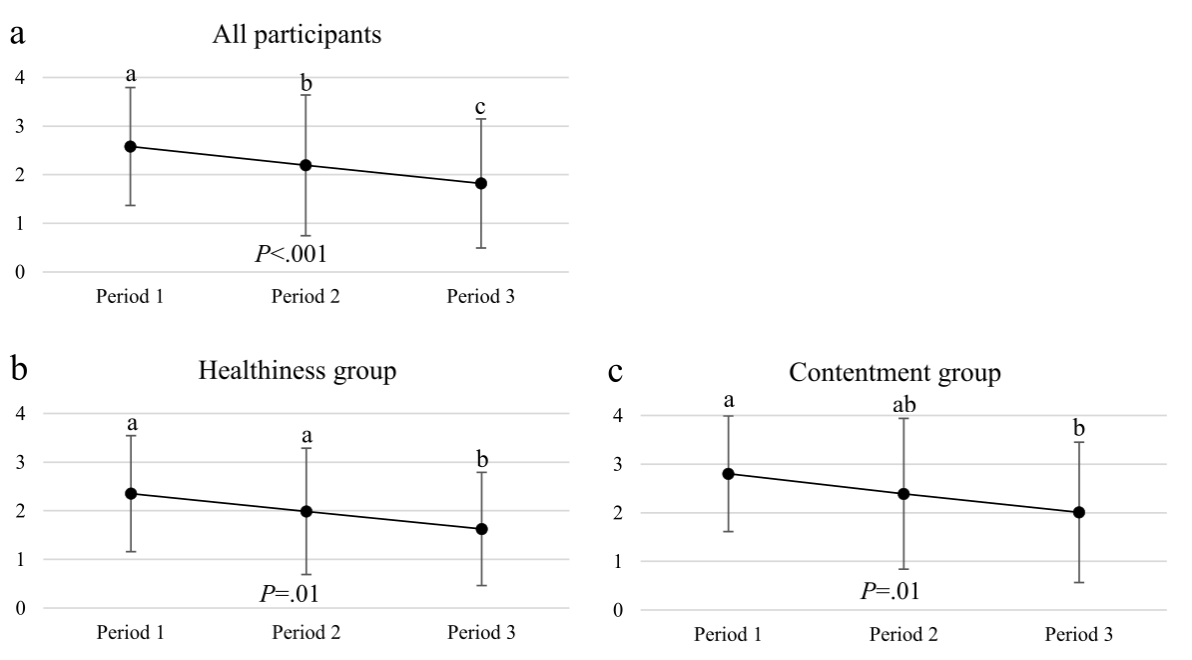
Adherence to usage of the Button application. The average number of times the application was opened per day during the three periods in a) all participants (n=59) and members of b) Healthiness group (n=29) and c) Contentment group (n=30).

Adherence to use the Button app decreased during the study among all participants. They opened the app to observe the visual summaries related to eating rhythm on average 2.6±1.2 (Period 1); 2.2±1.4 (Period 2), and 1.8±1.3 (Period 3) times per day (Figure 5). Within study groups, the trend was similar, but significant differences were observed only between Period 1 and Period 3. Between-group analysis showed no significant differences between the groups (Period 1: *F*_*1*_=2.042, *P=*.16; Period 2: *F*_*1*_=1.126, *P=*.29; and Period 3: *F*_*1*_=1.207, *P=*.28).

### Eating Behavior Tendencies

There were no differences between groups regarding cognitive restraint, uncontrolled eating, or emotional eating before the intervention (*F*_*1*_=.039, *P*=.85; *F*_*1*_=.177, *P*=.68; and *F*_*1*_=.900, *P*=.35, respectively). The reported tendencies of emotional eating, uncontrolled eating, and cognitive restraint were higher after the intervention than before the intervention in both the intervention groups (Table 4). The changes were statistically significant except that of uncontrolled eating among the members of Healthiness group. There were no differences between the groups in any of the measured eating behavior tendencies after the intervention (cognitive restraint: *F*_*1*_=.004, *P*=.95; uncontrolled eating: *F*_*1*_=.255, *P*=.62; emotional eating: *F*_*1*_=.493, *P*=.49).

**Table 4:** Participants’ (all participants and subgroups) responses to the three-factor eating questionnaire R15 (mean, SD, scale 0-100, in which higher score means greater tendency toward the measured subscale) before the intervention and after the intervention.

Eating behavior tendency		Mean (SD)		*F* test (*df*)	*P* value
		Before	After		
**Cognitive restraint**					
	All participants (N=74)	29.1 (23.5)	37.4 (20.9)	12.641 (*1*)	.001
	Healthiness group (n=37)	29.7 (28.5)	37.2 (24.2)	4.954 (*1*)	.03
	Contentment group (n=37)	28.6 (17.4)	37.5 (17.3)	7.781 (*1*)	.01
**Uncontrolled eating**					
	All participants	24.5 (13.2)	28.7 (13.6)	10.847 (*1*)	.002
	Healthiness group	25.2 (12.9)	27.9 (12.3)	1.912 (*1*)	.18
	Contentment group	23.9 (13.6)	29.5 (14.9)	12.532 (*1*)	.001
**Emotional eating**					
	All participants	20.7 (21.5)	29.7 (25.7)	20.785 (*1*)	<.001
	Healthiness group	18.3 (21.3)	27.6 (26.8)	11.833 (*1*)	.001
	Contentment group	23.5 (21.9)	31.8 (24.7)	8.925 (*1*)	.01

### Discontent With Eating

The participants were asked to evaluate how often they were discontent with their eating habits (1=never to 5=very often), and the average value for discontent was calculated for each meal time. In general, the participants were content with their eating habits (1.6-2.8, Table 5). The 2 groups did not differ regarding the frequency of discontent before the intervention (*F*_*1*_=1.303, *P*=.26; *F*_*1*_=.215, *P*=.64; *F*_*1*_=1.435, *P*=.24; *F*_*1*_=.889, *P*=.35; *F*_*1*_=.136, *P*=.71; *F*_*1*_=.014, *P*=.91 during breakfast time, lunch time, afternoon, dinner time, late evening or in the night, or some other time, respectively). More frequent discontent with eating habits during lunch time was reported after the intervention than before the intervention among all participants and among the members of the Healthiness group. There were no differences between the groups regarding the frequency of discontent after the intervention (*F*_*1*_=1.323, *P*=.25; *F*_*1*_=1.432, *P*=.24; *F*_*1*_=1.462, *P*=.23; *F*_*1*_=.543, *P*=.46; *F*_*1*_=.809, *P*=.37; *F*_*1*_=1.889, *P*=.17) during breakfast time, lunch time, afternoon, dinner time, late evening or in the night, or some other time, respectively.

**Table 5:** Participants’ (all participants and subgroups) evaluations of discontent with their eating habits (scale 1-5, in which 1=never, 5=very often; mean, SD) in different meal times before and after the intervention.

Meal times		Mean (SD)			*F* test (*df*)	*P* value
		Before		After		
**Breakfast**						
	All participants (N=74)	2.0 (1.0)		2.1 (0.9)	.451 (1)	.50
	Healthiness group (n=37)	2.1 (1.2)		2.2 (1.0)	.130 (1)	.72
	Contentment group (n=37)	1.9 (0.9)		2.0 (0.8)	.354 (1)	.56
**Lunch time**						
	All participants	2.0 (0.7)		2.2 (0.7)	10.790 (1)	.002
	Healthiness group	2.0 (0.8)		2.3 (0.8)	6.640 (1)	.01
	Contentment group	1.9 (0.7)		2.1 (0.6)	4.083 (1)	.05
**Afternoon**						
	All participants	2.6 (0.9)		2.7 (0.8)	1.820 (1)	.18
	Healthiness group	2.7 (0.9)		2.8 (0.8)	.722 (1)	.40
	Contentment group	2.5 (0.8)		2.6 (0.7)	1.090 (1)	.30
**Dinner time**						
	All participants	2.6 (0.9)		2.6 (0.8)	.899 (1)	.35
	Healthiness group	2.7 (0.9)		2.7 (0.7)	.163 (1)	.69
	Contentment group	2.5 (0.8)		2.6 (0.9)	1.000 (1)	.32
**Late evening or night**						
	All participants	2.6 (0.9)		2.6 (1.0)	.102 (1)	.75
	Healthiness group	2.6 (1.0)		2.5 (1.1)	.342 (1)	.56
	Contentment group	2.5 (0.9)		2.7 (1.0)	1.202 (1)	.28
**Some other time**						
	All participants	1.6 (1.0)		1.8 (0.9)	1.392 (1)	.24
	Healthiness group	1.6 (1.0)		2.0 (1.0)	1.891 (1)	.18
	Contentment group	1.7 (1.0)		1.7 (0.8)	.000 (1)	>.99

### Insights Into Eating Patterns

The participants reported that they had obtained insights into their eating patterns during the intervention period. These were related to eating rhythm, variation in eating rhythm, and healthiness of eating habits (Table 6).

### Attitudes and Motives

The measured attitudes toward health (General Health Interest) did not differ between the groups before the intervention (*F*_*1*_=.571, *P*=.45), and they did not change during the intervention (Table 7). The groups did not differ regarding attitudes toward health after the intervention either (*F*_*1*_=.406, *P*=.53).

The groups were similar regarding motives related to food choices before the intervention (Health: *F*_*1*_=.002, *P*=.97; Mood: *F*_*1*_=.000, *P*>.99; Weight Control: *F*_*1*_=.975, *P*=.33). Motives related to food choices remained unchanged within all the participants and subgroups except the increase in weight control motive in the Contentment group. No differences between the groups were identified after the intervention (Health: *F*_*1*_=.017, *P*=.90; Mood: *F*_*1*_=.426, *P*=.52; Weight Control: *F*_*1*_=2.629, *P*=.11).

**Table 6:** Examples of comments of the participants after Button usage in 3 main categories: eating rhythm, variation in eating rhythm, healthiness of eating habits, and their subcategories.

Main category	Subcategories and examples of comments (in brackets)
Eating rhythm	Observations on eating rhythm in relation to own preconceptions: “My eating rhythm was surprisingly regular even though I felt that I eat very irregularly”; Attention paid and observations made on one’s own eating rhythm: “Irregularity of my eating rhythm was shaped”; Observation of a relationship between eating rhythm, food choices, and wellbeing: “Regular eating maintains blood glucose levels (which I already knew) and I was more alert during the day (which I finally experienced concretely)”; Recognition of a need to change eating rhythm: “I am planning to reduce snacking”
Variation in eating rhythm	Observation of variation in eating rhythm because of the day of the week: “Eating rhythm is more irregular in weekends”; Observation of variation in eating rhythm because of time of the day: “I noticed that often during forenoon my eating rhythm is regular. Often, towards evening I either forget to eat or increase eating, depending on the day”; Observation of variation because of external factors: “There are workdays and travels during which I cannot ensure short enough gaps between meals without planning”
Healthiness of eating habits	Observation of healthiness of one’s own eating habits “In many days I eat some unhealthy snack”; Recognition of a need to improve eating habits: “I decided not to buy some ice cream and candy when I still saw the last red mark on the screen”

**Table 7:** Participants’ (all participants and subgroups) attitudes toward health (General Health Interest questionnaire, scale 1-7, in which 1=completely disagree, 7=completely agree) and motives related to food choices (Food Choice questionnaire, scale 1-4, in which 1=not important at all, 4=very important; mean, SD) before the intervention and after the intervention.

Participants’ attitudes and motives		Mean (SD)		*F* test (*df*)	*P* value
		Before	After		
**General Health Interest**					
	All participants (N=74)	5.0 (1.0)	4.9 (1.0)	1.039 (1)	.31
	Healthiness group (n=37)	4.9 (1.0)	4.9 (0.8)	.311 (1)	.58
	Contentment group (n=37)	5.1 (1.0)	5.0 (1.1)	.792 (1)	.38
**Health motive**					
	All participants	3.3 (0.4)	3.3 (0.4)	.047 (1)	.83
	Healthiness group	3.3 (0.4)	3.3 (0.5)	.004 (1)	.95
	Contentment group	3.3 (0.5)	3.3 (0.5)	.084 (1)	.77
**Mood motive**					
	All participants	3.1 (0.5)	3.1 (0.6)	1.578 (1)	.21
	Healthiness group	3.1 (0.5)	3.1 (0.5)	.079 (1)	.78
	Contentment group	3.1 (0.5)	3.1 (0.5)	2.614 (1)	.15
**Weight control motive**					
	All participants	2.5 (0.6)	2.6 (0.7)	2.807 (1)	.10
	Healthiness group	2.4 (0.7)	2.4 (0.7)	.111 (1)	.74
	Contentment group	2.5 (0.5)	2.7 (0.7)	4.751 (1)	.04

## Discussion

### Principal Findings

The 30-day self-monitoring of eating occasions with an EMA mobile phone app, *Button,* changed the eating patterns: the average interval between meal occasions lengthened and the number of daily-consumed meals decreased. The effectiveness of the app varied in the 2 study groups: the changes were statistically significant mainly among all the participants and among the members of Healthiness group. The participants reported higher tendencies of emotional eating, uncontrolled eating, and cognitive restraint after the intervention than before the intervention, and the discontent with eating slightly increased regarding eating at lunch time. Participants also reported having gained insights into the eating rhythm or eating habits during the intervention period, which indicates that the awareness on their eating patterns increased. The measured attitudes and motives remained unchanged except for a small increase in weight control motive among the participants of Contentment group. To sum up, the study results indicate that self-monitoring of eating occasions with an EMA tool might assist in battling against the growing tendency of irregular eating [9-13] and harms related to it [2,3,6-8]. The tool might be useful especially for people who follow a *grazing* eating pattern with no clear meal times but frequent peaks of eating occasions during the day. However, the underlying reasons for the more pronounced tendencies of emotional eating and uncontrolled eating after the intervention require further investigations.

The 2 Button versions were developed and studied among 2 similar study groups. The only difference between the versions was the logic of the 2 buttons (healthy-unhealthy, content-discontent), indicated by different button colors. The content-discontent version was intended to provide the users freedom and feeling of autonomy to choose whether they are content with the eating occasion regardless of its healthiness and therefore to motivate the users toward behavior change [20-23]. However, the observed changes in eating rhythm were statistically significant only in the Healthiness group, as opposed to the expectation. A possible reason for the result could be in the familiarity and more normative nature of the healthy-unhealthy concept, which might have made it easier to evaluate eating. Some indications about this were received during the usability studies carried out during the Button development in which healthy-unhealthy dichotomy was preferred by the participants because of its logical and easy-to-interpret nature. Both of the groups classified the majority of the eating occasions as *green* (healthy in Healthiness group, content in Contentment group). However, the proportion of red button presses (unhealthy) was larger than that of yellow button presses (discontent).

In both the study groups, the intensities of the 3 measured eating behavior tendencies (emotional eating, uncontrolled eating, and cognitive restraint) were elevated in postsurvey compared with presurvey. Cognitive restraint has been found to increase because of weight-loss interventions [32,33], whereas uncontrolled eating has been found to decrease among successful dieters [33,34]. Moreover, in a Web-based weight loss program, cognitive restraint increased and uncontrolled eating decreased among the participants who completed (620 out of 22,800 enrolled participants) the 6 months intervention [35]. However, eating behavior tendencies have found to change not only among intervention groups but also among control groups, indicating that they are not stable constructs [32,33].

High tendencies for cognitive restraint, emotional eating, and uncontrolled eating are considered to associate with generally negative phenomena such as high BMI and stress [36-38]. Therefore, the potentially adverse effects of self-monitoring of eating rhythm on these eating behavior tendencies cannot be ruled out. The increase in cognitive restraint could be regarded as a natural consequence of increased attention to eating, in accordance with earlier studies investigating the effects of dietary interventions on eating behavior tendencies [32,33]. However, the increases in emotional eating and uncontrolled eating are more cumbersome phenomena. In the case of cognitive restraint, it could be hypothesized that marking down the eating occasions might have strengthened the role of reflective cognitive processes instead of automatic processes that generally dominate dietary choices, meaning that increased attention and awareness of these tendencies might have influenced the evaluation [15]. After completing the questionnaire in the presurvey and paying attention to the eating pattern for 30 days, the participants might have had better capabilities to evaluate eating behavior tendencies in the postsurvey. This interpretation is supported by the open-ended responses related to insights into eating pattern during the intervention period. The majority of the participants reported that they had paid attention to their eating habits, and many of those insights were related to contrast between participants’ earlier beliefs and actual behavior illustrated by the app. These insights might have made participants more susceptible, precise, and realistic in their evaluations of eating tendencies in the postsurvey. However, as it is not clear if the increase in emotional eating and uncontrolled eating reflected actual changes in the eating behavior tendencies or just improved the ability to evaluate these tendencies, the potential adverse effects cannot be ruled out. Therefore, the evolvement of the eating behavior tendencies should be evaluated in future interventions.

The participants of both the intervention groups reported being more discontent with their eating during lunch time, after the intervention. This finding indicates that Button use made them more aware of lunch-time eating and the downsides of it (eg, inability to enjoy lunch break because of the hectic working pace or lack of good lunch options). In the open-ended questions, some participants shared this view. In a best-case scenario, the increased discontent might trigger changes in the lunch-time eating habits: more time could be preserved for lunch, or better lunch options could be sought. However, unless some changes can be made, the increased discontent can be interpreted as a negative effect of using the app.

A small increase in the weight-control motive was seen among the participants of the Contentment group, but there were no other changes in the food choice motives or attitudes. This is not surprising as attitudes are especially stable psychological constructs and difficult to be altered [39,40]. However, alteration of occasionally misrepresented beliefs behind the attitudes might be worthwhile as a change in those might in time lead to attitude change and eventually lead to behavior change [41,42]. In the case of this study, the responses to the open-ended questions suggested that the use of app was able to reveal to the participants some of their misrepresented beliefs related to eating behavior. Although this effect was not visible in attitude and motive measurements, it could be suggested that an app with more features, focusing on revealing incorrect beliefs might be powerful enough in time to alter even attitudes and thus result in long-lasting changes in eating patterns.

Self-monitoring of daily behavior is 1 way to become more aware of behavior, which in turn facilitates changes in behavior [15]. A majority of the apps for monitoring eating have been laborious to use, and adherence does not last long [43]. In this study, the participants opened the Button app to observe the summaries almost twice a day, even during the last 10 days of intervention. This indicates that adherence was relatively good, making the Button a feasible tool to monitor eating pattern.

### Limitations

The study has limitations. First, there was no control group without the Button app, which limits the interpretation of the results. Part of the observed changes might have occurred because of study participation rather than the use of the app itself. Moreover, we cannot rule out the possibility that some external factors, such as season of the year, could have contributed to the observed changes in the study. Control would have also been useful for evaluating how the repetition of TFEQ after a relatively short period (30 days) influences the results (ie, do the participants evaluate their tendencies differently after the first exposure to the questionnaire?). Second, the charm of the novelty of the app might have diminished toward the end of the intervention; therefore, the ease of remembering to mark every eating occasion might have weakened, influencing the number of daily presses. However, importantly, the reduction of daily consumed meals was observed in both survey data and data derived from the Button. Third, the intervention period of 30 days was relatively short. Engagement to the app use would have likely decreased over a longer period. However, we consider Button as a tool to become aware of temporal eating patterns rather than a tool for sustained use. Therefore, the period of 30 days is justified. However, a follow-up study would be especially useful to evaluate the long-term effects on eating behavior tendencies. Finally, Button data of 15 participants had to be excluded from the analyses. Complete case analysis was perceived as a more suitable approach than intention to treat with regard to Button data. The reasoning lies in the nature of the Button app, which is intended for voluntary use for those who are motivated to monitor their eating behavior. Therefore, including those who discontinued the app use might have led to very pessimistic results. On the other hand, complete case approach along with limited convenience sample might bias the results toward unjustified optimism. This bias is alleviated by the results derived from the survey data, which were based on the full sample. These results are in line with the observations from Button data. Considering the identified limitations, the future studies should include a control group, and there should be follow-up points to observe if the observed changes will sustain. The reported number of daily meals of the participants (4.5) was already as recommended in the Finnish nutrition recommendations—4 to 5 meals per day [44]—before the intervention. In future studies, it would be interesting to test the app among user groups who have a *grazing* meal pattern.

### Conclusions

The Button app was easy to use, and adherence was good. The results indicate that self-monitoring of eating with a simple mobile app may hold promise in promoting regular eating patterns. However, the suitability of the app for users with different meal patterns and eating behavior tendencies needs further studies.

## Abbreviations

ANOVA: analysis of variance
BMI: body mass index
EMA: ecological momentary assessment
FDR: false discovery rate
TFEQ: three-factor eating questionnaire

## References

[1] Kerver JM, Yang EJ, Obayashi S, Bianchi L, Song WO (2006). Meal and snack patterns are associated with dietary intake of energy and nutrients in US adults. J Am Diet Assoc.

[2] Leech RM, Worsley A, Timperio A, McNaughton SA (2015). Understanding meal patterns: definitions, methodology and impact on nutrient intake and diet quality. Nutr Res Rev.

[3] McCrory MA (2014). Meal skipping and variables related to energy balance in adults: a brief review, with emphasis on the breakfast meal. Physiol Behav.

[4] Berg C, Forslund HB (2015). The influence of portion size and timing of meals on weight balance and obesity. Curr Obes Rep.

[5] Leidy HJ, Campbell WW (2011). The effect of eating frequency on appetite control and food intake: brief synopsis of controlled feeding studies. J Nutr.

[6] Canuto R, da Silva Garcez A, Kac G, de Lira PI, Olinto MT (2017). Eating frequency and weight and body composition: a systematic review of observational studies. Public Health Nutr.

[7] Kärkkäinen U, Mustelin L, Raevuori A, Kaprio J, Keski-Rahkonen A (2018). Successful weight maintainers among young adults-A ten-year prospective population study. Eat Behav.

[8] Ekmekcioglu C, Touitou Y (2011). Chronobiological aspects of food intake and metabolism and their relevance on energy balance and weight regulation. Obes Rev.

[9] Pot GK, Almoosawi S, Stephen AM (2016). Meal irregularity and cardiometabolic consequences: results from observational and intervention studies. Proc Nutr Soc.

[10] Lund TB, Gronow J (2014). Destructuration or continuity? The daily rhythm of eating in Denmark, Finland, Norway and Sweden in 1997 and 2012. Appetite.

[11] Wittig F, Hummel E, Wenzler G, Heuer T (2017). Energy and macronutrient intake over the course of the day of German adults: A DEDIPAC-study. Appetite.

[12] Gill S, Panda S (2015). A smartphone app reveals erratic diurnal eating patterns in humans that can be modulated for health benefits. Cell Metab.

[13] Piernas C, Popkin BM (2010). Snacking increased among US adults between 1977 and 2006. J Nutr.

[14] Leech RM, Worsley A, Timperio A, McNaughton SA (2017). Temporal eating patterns: a latent class analysis approach. Int J Behav Nutr Phys Act.

[15] Burke LE, Wang J, Sevick MA (2011). Self-monitoring in weight loss: a systematic review of the literature. J Am Diet Assoc.

[16] Semper HM, Povey R, Clark-Carter D (2016). A systematic review of the effectiveness of smartphone applications that encourage dietary self-regulatory strategies for weight loss in overweight and obese adults. Obes Rev.

[17] Carter MC, Burley VJ, Nykjaer C, Cade JE (2013). Adherence to a smartphone application for weight loss compared to website and paper diary: pilot randomized controlled trial. J Med Internet Res.

[18] Franco RZ, Fallaize R, Lovegrove JA, Hwang F (2016). Popular nutrition-related mobile apps: a feature assessment. JMIR Mhealth Uhealth.

[19] Hand RK, Perzynski AT (2016). Ecologic momentary assessment: perspectives on applications and opportunities in research and practice regarding nutrition behaviors. J Nutr Educ Behav.

[20] Chia JL, Fuller-Tyszkiewicz M, Buck K, Chamari K, Richardson B, Krug I (2018). An ecological momentary assessment of the effect of fasting during Ramadan on disordered eating behaviors. Appetite.

[21] Levinson CA, Sala M, Fewell L, Brosof LC, Fournier L, Lenze EJ (2018). Meal and snack-time eating disorder cognitions predict eating disorder behaviors and vice versa in a treatment seeking sample: A mobile technology based ecological momentary assessment study. Behav Res Ther.

[22] Verstuyf J, Patrick H, Vansteenkiste M, Teixeira PJ (2012). Motivational dynamics of eating regulation: a self-determination theory perspective. Int J Behav Nutr Phys Act.

[23] Teixeira P, Patrick H, Mata J (2011). Why we eat what we eat: the role of autonomous motivation in eating behaviour regulation. Nutr Bull.

[24] Deci E, Ryan R (2000). The “What” and “Why” of goal pursuits: human needs and the self-determination of behavior. Psychological Inquiry.

[25] Ryan R, Patrick H, Deci E, Williams G (2008). Facilitating health behaviour change and its maintenance: interventions based on Self-Determination Theory. Eur Heal Psychol.

[26] Nunnally J, Bernstein I (1994). Psychometric Theory.

[27] Karlsson J, Persson LO, Sjöström L, Sullivan M (2000). Psychometric properties and factor structure of the Three-Factor Eating Questionnaire (TFEQ) in obese men and women. Results from the Swedish Obese Subjects (SOS) study. Int J Obes Relat Metab Disord.

[28] Pentikäinen S, Arvola A, Karhunen L, Pennanen K (2018). Easy-going, rational, susceptible and struggling eaters: a segmentation study based on eating behaviour tendencies. Appetite.

[29] de Lauzon B, Romon M, Deschamps V, Lafay L, Borys J, Karlsson J, Ducimetière P, Charles MA, Fleurbaix Laventie Ville Sante Study Group (2004). The Three-Factor Eating Questionnaire-R18 is able to distinguish among different eating patterns in a general population. J Nutr.

[30] Roininen K, Tuorila H, Zandstra E, de Graaf C, Vehkalahti K, Stubenitsky K, Mela D (2001). Differences in health and taste attitudes and reported behaviour among Finnish, Dutch and British consumers: a cross-national validation of the Health and Taste Attitude Scales (HTAS). Appetite.

[31] Konttinen H, Sarlio-Lähteenkorva S, Silventoinen K, Männistö S, Haukkala A (2013). Socio-economic disparities in the consumption of vegetables, fruit and energy-dense foods: the role of motive priorities. Public Health Nutr.

[32] Keränen A, Strengell K, Savolainen MJ, Laitinen JH (2011). Effect of weight loss intervention on the association between eating behaviour measured by TFEQ-18 and dietary intake in adults. Appetite.

[33] Nurkkala M, Kaikkonen K, Vanhala ML, Karhunen L, Keränen A, Korpelainen R (2015). Lifestyle intervention has a beneficial effect on eating behavior and long-term weight loss in obese adults. Eat Behav.

[34] Karhunen L, Lyly M, Lapveteläinen A, Kolehmainen M, Laaksonen DE, Lähteenmäki L, Poutanen K (2012). Psychobehavioural factors are more strongly associated with successful weight management than predetermined satiety effect or other characteristics of diet. J Obes.

[35] Svensson M, Hult M, van der Mark M, Grotta A, Jonasson J, von HY, Rössner S, Trolle LY (2014). The change in eating behaviors in a Web-based weight loss program: a longitudinal analysis of study completers. J Med Internet Res.

[36] Elfhag K, Linné Y (2005). Gender differences in associations of eating pathology between mothers and their adolescent offspring. Obes Res.

[37] Järvelä-Reijonen E, Karhunen L, Sairanen E, Rantala S, Laitinen J, Puttonen S, Peuhkuri K, Hallikainen M, Juvonen K, Myllymäki T, Föhr T, Pihlajamäki J, Korpela R, Ermes M, Lappalainen R, Kolehmainen M (2016). High perceived stress is associated with unfavorable eating behavior in overweight and obese Finns of working age. Appetite.

[38] Cornelis MC, Rimm EB, Curhan GC, Kraft P, Hunter DJ, Hu FB, van Dam RM (2014). Obesity susceptibility loci and uncontrolled eating, emotional eating and cognitive restraint behaviors in men and women. Obesity (Silver Spring).

[39] Eagly A, Chaiken S (1993). The Psychology Of Attitudes.

[40] Solomon M, Bamossy G, Askegaard S, Hogg M (2006). Consumer Behaviour: A European Perspective.

[41] Ajzen I, Fishbein M (1980). Understanding Attitudes and Predicting Behaviour.

[42] Malek L, Umberger WJ, Makrides M, ShaoJia Z (2017). Predicting healthy eating intention and adherence to dietary recommendations during pregnancy in Australia using the Theory of Planned Behaviour. Appetite.

[43] Reed JR, Struwe L, Bice MR, Yates BC (2017). The impact of self-monitoring food intake on motivation, physical activity and weight loss in rural adults. Appl Nurs Res.

[44] (2014). Finnish National Nutrition Council. Terveyttä ruoasta.

